# GD3 ganglioside is a promising therapeutic target for glioma patients.

**DOI:** 10.1093/noajnl/vdae038

**Published:** 2024-03-19

**Authors:** Victoria Hein, Nathalie Baeza-Kallee, Alexandre Bertucci, Carole Colin, Aurélie Tchoghandjian, Dominique Figarella-Branger, Emeline Tabouret

**Affiliations:** Aix-Marseille Université, CNRS, INP, Inst Neurophysiopathol, GlioME Team, Marseille, France; Aix-Marseille Université, CNRS, INP, Inst Neurophysiopathol, GlioME Team, Marseille, France; Aix-Marseille Univ, Réseau Préclinique et Translationnel de Recherche en Neuro-oncologie PETRA, Plateforme PETRA“TECH” and Plateforme PE”TRANSLA,” Marseille, France; Aix-Marseille Université, CNRS, INP, Inst Neurophysiopathol, GlioME Team, Marseille, France; APHM, CHU Timone, Service de Neuro-Oncologie, MarseilleFrance; Aix-Marseille Université, CNRS, INP, Inst Neurophysiopathol, GlioME Team, Marseille, France; Aix-Marseille Univ, Réseau Préclinique et Translationnel de Recherche en Neuro-oncologie PETRA, Plateforme PETRA“TECH” and Plateforme PE”TRANSLA,” Marseille, France; Aix-Marseille Université, CNRS, INP, Inst Neurophysiopathol, GlioME Team, Marseille, France; Aix-Marseille Univ, Réseau Préclinique et Translationnel de Recherche en Neuro-oncologie PETRA, Plateforme PETRA“TECH” and Plateforme PE”TRANSLA,” Marseille, France; Aix-Marseille Université, CNRS, INP, Inst Neurophysiopathol, GlioME Team, Marseille, France; Aix-Marseille Université, CNRS, INP, Inst Neurophysiopathol, GlioME Team, Marseille, France; APHM, CHU Timone, Service de Neuro-Oncologie, MarseilleFrance; Aix-Marseille Univ, Réseau Préclinique et Translationnel de Recherche en Neuro-oncologie PETRA, Plateforme PETRA“TECH” and Plateforme PE”TRANSLA,” Marseille, France

**Keywords:** Ganglioside, GD3, GD3 synthase, glioma, glioblastoma, stem cells

## Abstract

Glioblastoma is the most frequent and aggressive primary brain tumor in adults. Currently, no curative treatment is available. Despite first-line treatment composed by the association of surgery, radiotherapy, and chemotherapy, relapse remains inevitable in a median delay of 6 to 10 months. Improving patient management and developing new therapeutic strategies are therefore a critical medical need in neuro-oncology. Gangliosides are sialic acid-containing glycosphingolipids, the most abundant in the nervous system, representing attractive therapeutic targets. The ganglioside GD3 is highly expressed in neuroectoderm-derived tumors such as melanoma and neuroblastoma, but also in gliomas. Moreover, interesting results, including our own, have reported the involvement of GD3 in the stemness of glioblastoma cells. In this review, we will first describe the characteristics of the ganglioside GD3 and its enzyme, the GD3 synthase (GD3S), including their biosynthesis and metabolism. Then, we will detail their expression and role in gliomas. Finally, we will summarize the current knowledge regarding the therapeutic development opportunities against GD3 and GD3S.

**Key Points**

- GD3 is the major ganglioside of gliomas.
- GD3 is involved in the proliferation, migration, invasion, and stemness of glioma cells.
- Targeting GD3 and GD3 synthase is promising for glioma patients.

Glioblastoma is the most frequent and aggressive primary brain tumor for adults. To date, despite the combination of surgical resection, radiotherapy, and chemotherapy as the first-line treatment approach, there is currently no curative treatment available.^1^ Recurrence typically occurs within a median timeframe of 6 to 10 months, and the median overall survival following optimal first-line treatment is 14 months.^1^ Cytotoxic chemotherapy yields a response rate above 5% at relapse, while the monoclonal antibody Bevacizumab, targeting vascular endothelial growth factor, enhances the response rate to 40% during recurrence. However, it fails to improve overall survival in phase 3 trials at both initial diagnosis and relapse.^2,3^ Despite the transformative impact of targeted therapy and immunotherapies in systemic tumor treatments, these approaches have fallen short in various clinical trials dedicated to glioma patients.^4^ Therefore, the imperative lies in enhancing patient management and developing novel therapeutic strategies in neuro-oncology.

Gangliosides, sialic acid-containing glycosphingolipids, are highly prevalent in the nervous system,^5^ significantly contributing to cell glycocalyx and influencing cell surface features. They play a crucial role in modulating intricate interactions within the cellular membrane (*cis* interactions) or with soluble molecules in the extracellular environment and those associated with the surfaces of other cells (*trans* interactions; reviewed in^6,7^). During brain development, ganglioside expression patterns shift from simple ones like GM3 and GD3 to complex ones such as GM1, GD1a, and GD1b. GD3, a b-series ganglioside with 2 sialic acids, exhibits high expression in human embryonic neural stem cells but not in the normal adult brain.^8,9^ In cancers, GD3 is notably overexpressed in neuroectodermal-derived tumors like melanoma, neuroblastoma, and gliomas compared to its basal expression in most non-neoplastic tissues.^10,11^ Recent studies, including our own, have highlighted GD3’s direct role in glioma invasion, motility, tumor growth, and its implication in the stemness of glioblastoma cells, potentially contributing to gliomagenesis.^12,13^

This review provides a comprehensive overview of current knowledge on GD3 biosynthesis, expression patterns, its involvement in glioma initiation and progression, and promising future therapeutic developments.

## Review

### GD3 and GD3 Synthase in Physiological Conditions

#### Structure, biochemistry, synthesis, and O-acetylation of gangliosides.

**Figure 1.**
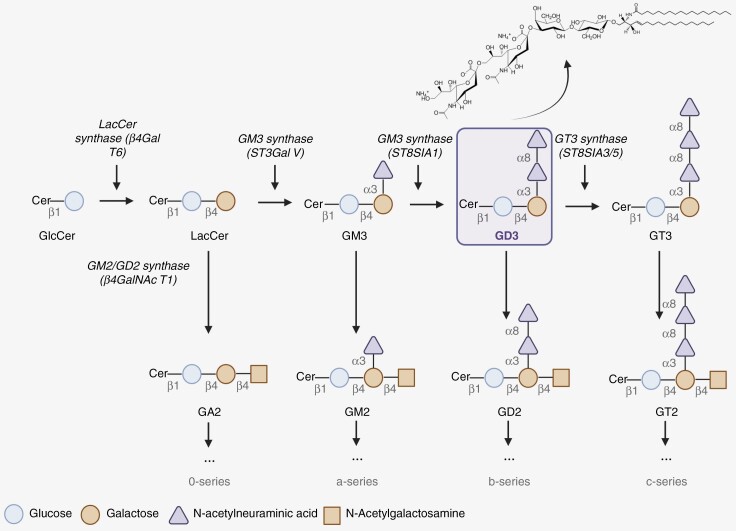
Part of the biosynthesis pathway for gangliosides, focusing on GD3. Biochemical formula of GD3 is shown (cited from: https://avantilipids.com/product/860060).

—The gangliosides constitute a subclass of membrane-bound amphipathic and acidic glycosphingolipids, composed by a common hydrophobic ceramide moiety, which acts as a membrane anchor, and a hydrophilic oligosaccharide chain, containing one or more sialic acid residues.( Figure 1) Sialic acid is exposed to the cellular environment functioning in intrinsic (*cis* interactions) and extrinsic (*trans* interactions) communication.^6,7^ Gangliosides are widely expressed mainly, but not exclusively, on the outer leaflet of the plasma membrane of most mammalian cells, according to a specific profile of the species, the tissue considered, and the stage of development or cellular differentiation. Thus, they interact with extracellular matrix, other membrane components such as phospholipids and cholesterol, and transmembrane proteins including receptors. They modulate vital processes such as cell growth, differentiation and adhesion, cell-to-cell recognition, cell signal transduction events, and cell death.^14^ Moreover, they have been found to be highly important in immunology. Gangliosides are not evenly distributed in the membrane but are rather clustered in lipid raft microdomains.^15^ It is noticeable that they are particularly abundant in the nervous system, the human brain containing 10- to 30-fold more gangliosides than any other tissue or organ in the body.^8^ The biological importance of gangliosides has been revealed by analyses of genetically engineered mice deficient in various ganglioside synthases. Mice suffered of motor and sensory dysfunctions, along with enhanced cell apoptosis, axonal degeneration, and perturbed axon-glia interactions in the cerebral cortex, leading in some cases to early death.^5^

Gangliosides are generally named according to the nomenclature described by Svennerholm,^16^ based on the order of migration in chromatography. The specific names for the gangliosides provide information about their structure. The letter “G” refers to ganglioside and the number of sialic acids is identified as “A” = 0, “M” =1, “D” = 2, “T” = 3, “Q” = 4. The numbered subscripts 1, 2, and 3 refer to the carbohydrate sequence that is attached to the ceramide. For example, “3” stands for GalGlc-ceramide.^17^ So far, more than 180 types of gangliosides have been identified in vertebrates.^5^

Gangliosides are first synthesized in the endoplasmic reticulum and the acceptor lipid molecule is further modified in the Golgi apparatus following stepwise action of various glycosyl- and sialyltransferases.^5^ Disturbance of the biosynthetic pathways leads to changes in the ganglioside profile and therefore to alterations in cellular metabolism and function.^18^ The ST8SIA (alpha-2,8-sialyltransferase) family catalyzes the transfer of sialic acid to another sialic acid in an α2,8-linkage.^19^ ST8SIA1, ST8SIA3, ST8SIA5, and ST8SIA6 are involved in the synthesis of oligosialic acid chains meaning that they display a chain of 2–7 sialic acids. On the other hand, ST8SIA2 and 4 are called polysialyltransferases because they participate in the elaboration of linear chains of 8 or more sialic acids.^20^

The b-series ganglioside GD3 structure was first identified in developing brain as consisting of ceramide, glucose, galactose, n-acetylneuraminic acid in molar ratios of 1:1:1:2. Ledeen et al. subsequently confirmed that this structure was identical to that of the ganglioside G3A, which levels were high in the brain of a patient with subacute sclerosing leukoencephalitis.^21^ The chemical formula of GD3 is C_72_H_129_N_3_O_29_ and according to Svennerholm’s nomenclature, it corresponds to alpha-n-Acetylneuraminyl-2,8-alpha-n-acetylneuraminyl-2,3-beta-d-galactosyl-1,4-beta-glucosyl-1,1’-ceramide.^16^ Some gangliosides, including GD3, can promote tumor-associated angiogenesis and strongly regulate cell adhesion, triggering tumor metastasis. Moreover, ganglioside antigens present on the cell surface act as immunosuppressors.^22^

One of the most common modifications of the sialic acid moiety described in nature is the formation of O-acetyl ester(s) at one or more of the hydroxyl group(s). This process is highly regulated and depends on species, tissue, cell type, and development stage. In particular, the proapoptotic effects of GD3 can be counteracted by 9-o-acetylation of GD3.^23^o-acetylation is an important and frequent modification of the ganglioside structure that results in the addition of an acetyl group on the 9-o-carbon position of the terminal sialic acid to form 9-o-acetyl GD3. Moreover, Birks et al. have described a critical ratio between GD3 and 9-o-acetyl GD3 (CD60b), which promotes tumor survival.^24^ Finally, the expression of the 9-o-acetyl esterase is sufficient to induce apoptosis of U87-MG glioblastoma cell line which expresses high levels of 9-o-acetyl GD3.^25^ Another interesting point regarding 9-o-acetyl GD3 is that in glioblastoma, it seems to be expressed intracellularly.^24^

#### Expression pattern of gangliosides in tissue and in the nervous system.

—Whereas GD3 is a minor ganglioside in most normal adult tissues, its expression increases during development and in pathological conditions such as cancer and neurodegenerative disorders.^25^

Under normal physiological conditions, gangliosides are widely expressed in mammalian tissues but are particularly abundant in the nervous system, where they were first isolated and characterized.^5^ Gangliosides promote the maturation of the nervous system^6^ and are mainly found on the surface of neuronal cells where they play essential roles in maintaining axon-myelin stability and function.^14^ Because of their spatio-temporal expression patterns, GD3 and the A2B5 epitope of c-series gangliosides have been considered as specific markers of early brain development.^26^ In the early mammalian embryonic brain, including retina and cerebellum, the pattern of ganglioside expression is limited to simple gangliosides, predominantly GM3 and GD3.^9^ Both have been associated with important developmental processes such as cell migration, axonal extension, and participate in the regulation of synapse plasticity. Furthermore, GD3 is thought to play a crucial role in maintaining the self-renewal capacity of neural stem cells, and thus neurogenesis, by supporting EGF signaling pathway, as demonstrated in mouse by Wang et al.^27^ This leads to the activation of cell proliferation which is essential for the self-renewal of stem cells in the brain.

At later developmental stages, however, more complex gangliosides predominate, in particular GM1, GD1a, GD1b, and GT1b until they constitute more than 90% of the total ganglioside mass in adults.^9^ This evolution is linked to developmental changes in the activity and gene expression of glycosyltransferases, regulated specifically to the cell stage and type, both at the transcriptional and post-translational levels.^28,29^

#### Subcellular location and expression of the GD3 synthase.

—GD3 synthase (GD3S), also known as ST8SIA1, for ST8-Alpha-n-acetyl-neuraminide Alpha-2,8-Sialyltransferase 1, is identified under the code EC 2.4.3.8 according to the revised international numerical classification of enzymes (KEGG Enzyme Nomenclature). It is a type II membrane protein of 356 amino acids (40519 Da), observed in the Golgi apparatus, that catalyzes the transfer of a sialic acid residue from CMP-sialic acid *via* an alpha-2,8-linkage onto GM3 to give rise to the disialoganglioside GD3.^30^ GD3 serves as a precursor to more complex gangliosides as it can be further processed to give rise to b- (GD2) and c-series (GT3) gangliosides.

The amino acid sequence of GD3S contains a single transmembrane domain in its n-terminal region and shows a high level of similarity to other sialyltransferases at 2 conserved regions. The human chromosomal location of the *GD3S* gene (ENSG00000111728) was determined by fluorescence in situ hybridization and mapped to 12p12.1–p11.2 locus.^30,31^ Expression of the *GD3S* mRNA (9707 bp, RefSeq NM_003034) among normal human tissues was reported to be highly restricted to fetal and adult brains and to a lesser extent in fetal and adult lungs.

### Expression and Role of GD3 and GD3S in Diffuse Gliomas

#### Tissue expression of GD3 and GD3S in diffuse gliomas.

##### Tumor tissue distribution.

The ganglioside GD3 was reported to be one of the major gangliosides expressed by gliomas and more specifically by glioblastoma.^10,32–36^ By using a mass spectrometry characterization, Fabris et al. observed that GD3 accounted for more than 50% of the total ganglioside content in glioblastoma and showed that the most abundant ion in glioblastoma corresponded to GD3 ganglioside with d18:1/18:0 ceramide composition, followed by GD3 containing d18:1/24:0 and d18:1/24:1 ceramides.^33^

GD3 expression was reported within sub-populations of human glioblastoma tumor cells,^37^ with expression variability across the different tumor areas.^37^ Indeed, O’Neill et al., observed intra-tumor and inter-patient GD3 expression heterogeneity through a mass spectrometry imaging approach.^37^ A higher expression of GD3 was observed in the central part of the tumor, associated with proliferation and angiogenesis,^38^ whereas it was less frequently expressed in the peripheric areas.^39,40^ In this line, GD3 was intensively expressed in hyper-vascularized areas,^41^ where they participated in the stem cell niche constitution. Nevertheless, GD3-positive cells were detected in the peritumoral tissue up to 3.5 cm from the tumor edge, suggesting their implication in glioblastoma progression and invasion.^39^ In contrast, GD3 was reported to be absent or extremely low in normal human brain,^42,43^ reinforcing its interest as therapeutic target.

GD3S is the only enzyme that regulates the biosynthesis of GD3.^12,13,44^ Recently, it has been reported that *GD3S* gene expression was regulated by DNA methylation at the promoter regions in gliomas, highlighting the role of *GD3S* epigenetic profile in the regulation of glioma phenotype and malignancy.^45^ Regarding the glioma cell distribution of GD3, it was mainly located in clusters on the cell surface, but it was also observed in the cytoplasm of tumor cells. Beyond glioma cells,^38,39,46^ GD3 was also expressed in endothelial cells, pericytes, or reactive astrocytes surrounding the tumor mass but did not seem to be expressed by normal astrocytes, limiting the risk of inhibitor toxicity. Nevertheless, expression of GD3 in the tumoral microenvironment was suggested to remain lower than in glioma or glioblastoma cells.^39^

Finally, 2 other expression profiles are currently lacking in the literature. First, the analysis of *GD3S* expression using single-cell RNA sequencing approach would be very helpful in discriminating the GD3 expression pattern across different tumor cell subtypes and microenvironment cell subsets. Secondly, the evaluation of GD3 expression at recurrence, after radio-chemotherapy for example, would be relevant to validate the potential role of GD3 in treatment resistance and tumor recurrence. For now, no study is available on these topics, but these analyses are ongoing in different teams, including ours.

##### Tumor grade and prognosis.

The prognostic impact of GD3 and *GD3S* expression in gliomas remains debated. The current data regarding GD3 expression and glioma grade are numerous and strong. In contrast, the data concerning GD3 expression and survival remain weak.

Hence, it has been shown that the concentration of GD3 in human glioma tissues was strongly correlated with the tumor grade in different studies.^38,47–49^ Similarly, the *GD3S* expression correlated with the astrocytoma grades.^13^ Indeed, a higher grade was associated with an increase in GD3 amount and with a decrease in all other ganglioside ratios.^10,50–52^ This GD3 increase was suggested to be almost specific of gliomas, compared to other brain tumors.

In contrast, the correlation between GD3 expression and patient survival has been less studied. Lama et al., analyzed the expression of GD3 in 36 patients and observed no correlation between GD3 expression level and patient survival.^39^ Nevertheless, based on the biological implication of GD3 in gliomagenesis (see below), survival impact needs to be explored in larger prospective cohorts and correlated to other prognostic factors and treatment response.

#### GD3 expression in liquid biopsies of patients with gliomas.

—GD3 expression was also analyzed in serum and cerebrospinal fluid (CSF) samples of patients with glioma and compared to those of healthy donors.^53^ GD3 was not detected in healthy donors or patients with low-grade glioma sera but it was detected in the sera of 30% of grade 3 astrocytoma patients and almost 80% of glioblastoma patients. Moreover, Radic et al. analyzed the GD3 serum level before and after surgical resection of glioma and observed a post-operative decrease of GD3 level in the sera samples collected from patients with complete tumor removal.^54^

In the CSF, Ladisch et al.,^55^ observed a significant increase of GD3 in patients with primary brain tumors, supporting the concept of ganglioside shedding into the CSF. However, because of the low number of samples, they were not able to confirm the prognostic value of this CSF expression, as well as its predictive value of response during the course of the tumor treatment.

#### Implication of GD3 and GD3S in glioma oncogenesis.

**Figure 2.**
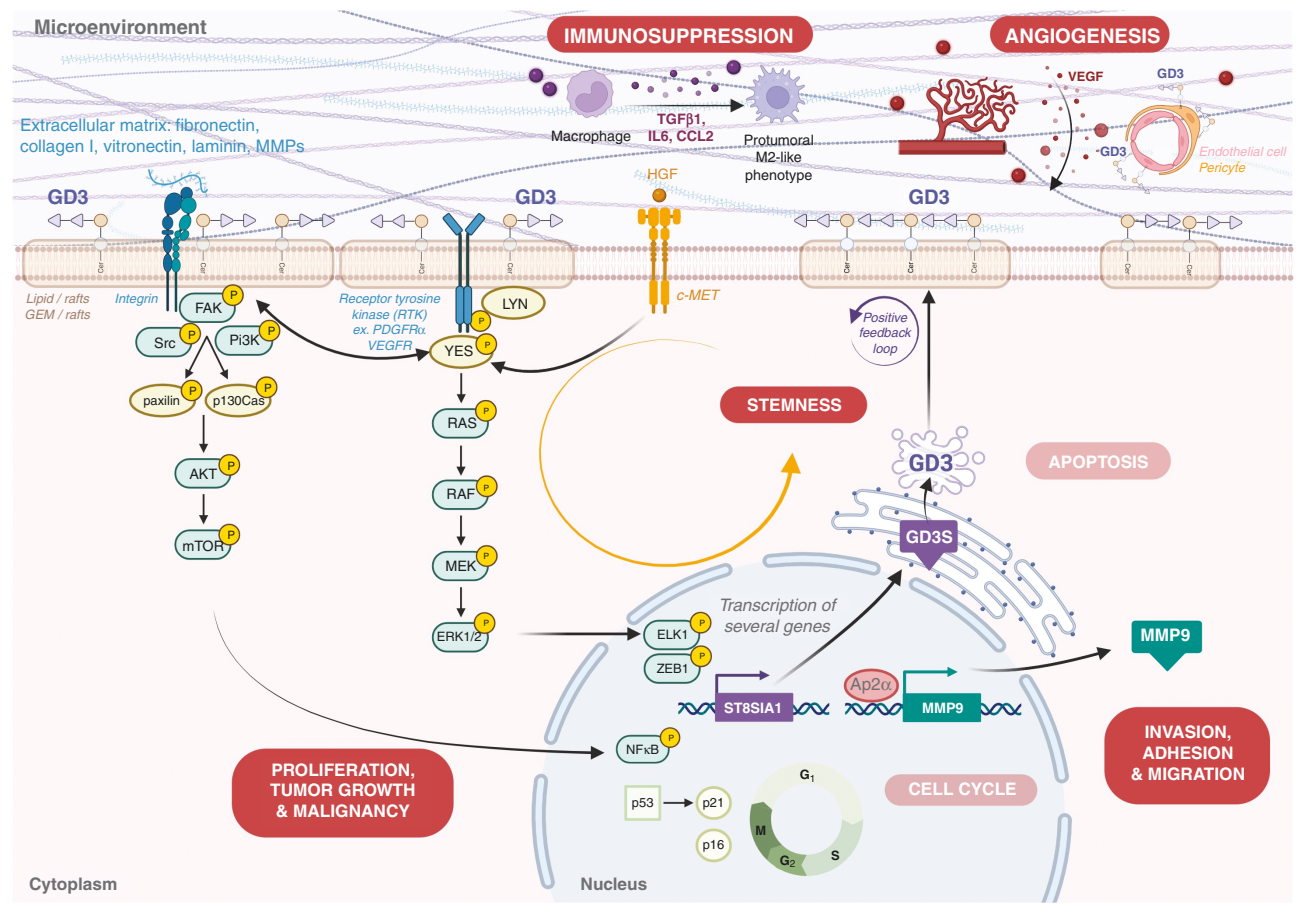
Main roles of GD3 and GD3 synthase in glioma cells and their interactions with the microenvironment.

—Different publications highlighted the implication of GD3 and *GD3S* in glioma cell proliferation, stemness, migration, invasion, apoptosis, but also in tumor growth, cell cycle regulation, and tumor microenvironment. (Figure 2) If several results are provided by in vitro experiments, strong results are reported by the team of Furukawa^12,56–58^ and their very interesting *GD3S* knockout mouse model, but also by the study of Yeh et al., showing the impact of GD3 on glioblastoma tumorigenicity.^13^

##### Proliferation and tumor growth.

GD3 was reported to be involved in glioma cell proliferation and tumor growth. First, a higher expression of GD3 was reported in the tumor proliferative area, supporting its involvement in glioma tumor growth.^11,38,59^ Moreover, in preclinical models, Ohkawa et al. showed that a depletion of *GD3S* was associated with a significantly longer survival of mouse models.^56,57^ These mice presented smaller gliomas with oligodendroglioma-like features, whereas those in control mice showed glioblastoma-like features after pathological analyses.^56,58^ These results were in line with other preclinical studies reporting that tumor cells expressing a high level of GD3 induced more aggressive tumors, growing faster, and with an increased proliferation rate.^60^

This impact on tumor cell proliferation may be related to the activation of several oncogenic pathways, including the MAP Kinase pathway. In glioma tissue, the phosphorylation levels of the AKT serine/threonine kinase family, the src family kinases and the extracellular signal-regulated kinases (ERK) were significantly lower in GD3S-KO cells than in control conditions.^12,57^ Moreover, it was reported that GD3 was able to form molecular complexes with membrane molecules such as platelet-derived growth factor receptor PDGFRα^57^ or the SFK Lyn,^61^ promoting malignant properties of glioma cells by enhancing cell signals transduced through membrane microdomains (lipid/rafts) and then proliferation signals. Thus, GD3 is probably able to recruit mutated receptor tyrosine kinases to glycolipid-enriched microdomain GEM/rafts and to activate these receptor tyrosine kinases in a ligand-independent manner, leading to the increased malignant properties of gliomas. On the other hand, it was suggested that activated ZEB1 and ELK1 by RTK/RAS/PI3K signals may enhance the expression of GD3 by forming a positive feedback loop.^44^

##### Apoptosis and cell cycle.

The implication of GD3 in the apoptosis process of glioma cells remains unclear and debated. If cytoplasmic GD3 showed proapoptotic activities in vitro,^62^ these results were in contradiction with the pro-tumoral activity of GD3 on proliferation and tumor cell survival. This duality could be explained by different observations. First, the proapoptotic role of cytoplasmic GD3 in glioma cells was mainly observed in one glioblastoma cell line, the U-1242 MG, and needs to be confirmed with more extensive observations.^62^ Secondly, the apoptotic implication of GD3 was reported to be related to the acetylation balance of cytoplasmic GD3 and notably its o-acetylation, which is considered to have an antagonist effect on GD3 function and to modulate its activities.^25^ 9-o-acetylGD3 was reported to be completely ineffective in inducing cytochrome c release and caspase-9 activation and failed to induce the collapse of mitochondrial transmembrane potential and cellular apoptosis in glioma cells.^25^ In this line, Birks et al.^24^ reported a critical ratio between GD3 and 9-o-acetyl GD3 in the cytoplasm of glioblastoma cells, in favor of cell survival and apoptosis resistance. Nevertheless, if the proapoptotic impact of GD3 in glioma cells seems to be limited, GD3 and 9-o-acetyl GD3 do not promote apoptosis resistance. Iwasawa et al.^12^ reported the absence of difference in apoptosis rate between GD3 positive and negative glioblastoma cell lines. In contrast, they observed a significant modification of cell cycle repartition according to GD3 expression level. By comparing the cell cycle repartition between GD3 negative and positive cells, the ratio of GD3 negative cells in G1 phase was significantly higher than the positive GD3 glioblastoma cells. In turn, the numbers of GD3 negative cells in G2/M and S phases were significantly lower than the GD3 positive cells. The authors confirmed these results by western blot and observed an accumulation of p16 and p21 in the GD3 negative cells as well as an increased phosphorylation of p53, leading to cell cycle arrest in GD3 negative cells.

##### Invasion, adhesion, and migration.

GD3 has also been involved in glioma cell motility and invasion.^11,39,60^ During the invasion process, GD3 was reported to be upregulated, facilitating tumor cell adhesion to various extracellular matrix components, such as integrins, laminin or matrix metalloproteinases, and influencing their expression, secretion, and functions.^11,63–65^ First, GD3 was reported to be the most effective adhesion-promoting ganglioside in all glioma cell lines tested on fibronectin collagen I, vitronectin, and laminin. GD3 was also reported to be involved in matrix metalloproteinases (MMP) expression.^63^ After analyzing the mRNA expression profile of *GD3S*-KO-generated tumors, Ohkawa et al. observed a significant downregulation of MMP family genes. Notably, they showed that this downregulation was impaired by the reintroduction of GD3S, with a specific increase of MMP9, led by the transcription factor activator protein 2α (Ap2α).^56^ Moreover, the involvement of GD3 and *GD3S* in the adhesion and invasion of glioma cells is also related to non-receptor tyrosine kinases (NTKs) such as focal adhesion kinase, or NTKs substrates such as paxillin or p130Cas, with an increasing phosphorylation rate of these proteins in positive-GD3 cells versus negative-GD3 cells.^12^ Finally, it has been reported that GD3 enhanced PDGFRα-mediated signals by forming a ternary complex consisting of GD3, PDGFRα, and Yes kinase (a non-receptor protein tyrosine kinase that belongs to the SFK family) in lipid rafts of the plasma membrane, leading to an increased invasiveness of glioma cells in vitro.^57^ This invasion activation by Yes kinase occurs through the activation of paxillin.

##### Tumorigenesis and stem cells.

It is now admitted that bulk glioblastoma tumors harbor cancer stem cells, a distinct subpopulation of cancer cells which are able to initiate new tumor after serial transplantation, have a long-term self-renewal capacity, and are resistant to radio-chemotherapy driving relapses.^66^ Various authors, including us, observed a high GD3 expression in stem-like cells sampled from glioblastoma bulk.^39,67^ Moreover, these cells also expressed a high amount of *GD3S*. One major study in this field was published by Yeh et al. in 2016.^13^ After generating tumorospheres from commercial glioblastoma cell lines in a serum-free medium containing EGF and basic FGF, the authors showed that the cells with high GD3 expression displayed functional characteristics of glioblastoma stem cells. The GD3-high cells showed self-renewal ability with expression of stemness genes and higher propensity to form secondary tumorospheres. Moreover, these cells were capable of tumor initiation in mouse models, suggesting that GD3 was a major determinant of glioblastoma tumorigenicity. In contrast, the downregulation of *GD3S* suppressed tumorosphere formation, led to the downregulation of stemness genes, and significantly reduced tumor initiation and growth in vivo. Finally, the authors showed that the implication of *GD3S* in stemness was mediated through c-Met signaling pathway activation.

##### Tumor microenvironment.

GD3 was reported to be strongly involved in tumor angiogenesis. GD3 was intensively expressed in hyper-vascularized areas of high-grade gliomas^51^ and was reported to be expressed not only by tumor cells but also by endothelial cells and pericytes in glioblastoma tissue.^39^ In contrast, a reduced density of vessels was observed in an inducible glioma *GD3S*-downregulated models, highlighting the implication of GD3S and GD3 in glioblastoma vessel proliferation and angiogenesis.^58^ The implication of GD3 in this process was suggested to be notably related to its ability to facilitate and enhance the release of vascular endothelial growth factor by glioblastoma cells.^68^

GD3 also displays immunosuppressive effects inhibiting host antitumor response. By using a *GD3S*-KO mouse model in an in vivo model of inducible glioma RCAS/Gtv-a system, Zhang et al.^58^ observed that reactive microglia/macrophages showed different localization patterns between *GD3S*-KO and control mice. CD69 + and Iba1 + cells were more frequently located inside glioma tissues in *GD3S*-KO mice, while they were located mainly around glioma tissues in control mice. Moreover, the number of CD68 + and Iba1 + cells significantly increased in the tumor tissue of *GD3S*-KO mice and these cells expressed a higher level of inducible nitric oxide synthase in favor of an anti-tumoral M1 phenotype. Finally, they observed a lower expression of pro-tumoral cytokines like TGF-β1, CCL2, or IL6 in the *GD3S*-KO mice. Taken together, these interesting results suggest that GD3 and *GD3S* allow glioma cells to promote polarization of microglia/macrophages towards a pro-tumoral M2-like phenotype by modulating the expression of chemokines and cytokines in brain tumor microenvironment.

### Therapeutic Development Opportunities to Target GD3 and GD3S

**Figure 3.**
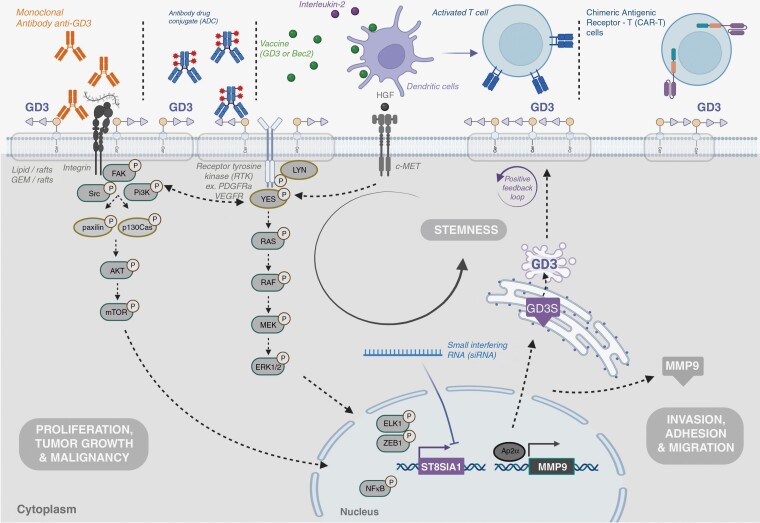
Different therapeutic strategies targeting GD3 or GD3 synthase have already been developed in preclinical or clinical settings.

Development of therapies targeting the ganglioside GD3 and the GD3S started almost 30 years ago. Different therapeutic approaches were developed, alone or in combination, including direct inhibition with monoclonal antibodies or by stimulating the immune response with vaccines. Currently, no clinical development targeting GD3 or GD3S is ongoing in the neuro-oncology field. Here, we reviewed the available clinical studies addressing anti-GD3 or GD3S treatments in oncology (Figure 3, Table 1).

**Table 1.** Summary of Clinical Trials Targeting GD3

Trial	Phase	Disease	Population	*N*. of patients	Treatment	PFS	OS	Other
Ragupathi et al. ^69^	I	Melanoma	After complete resection	12	GD3-L-KLH + QS-21	/	/	58% developed IgG anti-GD3 Ab
Yao et al. ^70^	I	Melanoma	After complete resection	18	Bec2-KLH-BCG	/	/	0% developed IgG anti-GD3 Ab
Chapman et al. ^71^	II	Melanoma	After complete resection/ high-risk	50	Bec2	16	50	64% developed anti-Bec2 Ab
Chapman et al. ^72^	II	Melanoma	After complete resection	24	GD3-L-KLH + QS-21 or Bec2	22	45	42% developed anti-GD3 Ab
Giaccone et al. ^73^	III	SCLC	Localized after CTRT	515	Bec2-BCG vs. follow-up	/	14.3 vs. 16.4	36% Grade 3 skin reaction
Cheung et al. ^74^	II	Neuroblastoma	High-risk	102	OPT-821 b-glucan	PFS 45.3% at 2 years	88.4% at 5 years	0% anti-GD3 Ab only anti-GD2
Rosenbaum et al. ^75^	II	Sarcoma	After metastasis resection	136	OPT-821 +/− KLH	34.5% vs. 34.8%	93.1% vs. 91.5%	15% SAEs
Scott et al. ^76,77^	I	Melanoma	Metastatic	17	KM-871	/	/	1PR/2SD
Tarhini et al. ^78^	I/II	Melanoma	Metastatic	36	KW-2871+ HD IFNa2b	PFS 2.5	10.3	/
Houghton et al. ^79^	I	Melanoma	All stages	12	R24	/	/	5PR
Maguire et al. ^80^	I	Melanoma	Metastatic	11	R24	/	/	0PR
Kirkwood et al. ^81^	Ib	Melanoma	Metastatic	37	R24	/	/	1CR/1PR
Bajorin et al. ^82^	I	Melanoma	Metastatic	20	R24 + IL-2	/	/	1PR
Soiffer et al. ^83^	Ib	Melanoma	Metastatic	28	R24 + IL-2	/	/	1PR
Minasian et al. ^84^	Ib	Melanoma	Metastatic	8	R24 + TNFa	/	/	6PR
Alpaugh et al. ^85^	Ib	Melanoma	Metastatic	20	R24 + IL-2 + TNFa	/	/	0 response
Minasian et al. ^86^	I	Melanoma	Metastatic	9	R24 + M-CSF	/	/	3PR

OS, overall survival; L, lactone; KLH, keyhole limpet hemocyanin; Ab, antibody; BCG, Bacille Calmette-Guerin; CTRT, chemoradiotherapy; PFS, progression-free survival; SAEs, serious adverse events; PR, partial response; SD, stable disease; CR, complete response; IFN, interferon; IL, interleukin; TNFa, tumor necrosis factor alpha.

Regarding the safety of GD3 inhibition, the predicted toxicities appear acceptable. The *GD3S* knockout mouse model did not show significant toxicity: Mouse models showed no abnormal behavior and no modification in hematological subsets.^18^

#### Immunotherapies.

—Around the 2000s, vaccinal strategies that aimed to obtain anti-GD3 immunization have been developed mainly in malignant melanoma or other solid tumors.^87^ The main immunotherapies developed against GD3 were an anti-idiotypic antibody, Bec2 (xenogeneic protein)-keyhole limpet hemocyanin-adjuvant (KLH).

First, the evaluation of a vaccine with conjugation of GD3-lactone with KLH was associated with immunogenic response in 12 patients with melanoma, and all patients with a saponin immunologic adjuvant, QS-21 (*n* = 6), developed antibodies against GD3.^69^ Another approach corresponded to the use of Bec2, that appeared to mimic GD3 and could induce anti-GD3 antibody response in patients. Bec2 vaccination was tested with or without KLH. In a phase 1 clinical trial, patients with melanoma (*n* = 18) received Bec2-KLH at 2.5 mg for intradermal immunization after complete surgical resection and Bec2 unconjugated at 10mg intravenously to boost the immunological response. Four patients developed IgM anti-GD3 but no patient developed IgG anti-GD3, despite the use of the immunization booster.^70^ Unfortunately, this association showed no impact on immunization.^70^ The association of Bec2-KLH with Bacille Calmette-Guerin (BCG) therapy showed promising results in inducing anti-GD3 antibodies for 3 out of 14 patients with melanoma after complete resection and at high risk of relapse in a phase 1 clinical trial. In this study, another immunological adjuvant QS-21 was tested but no patient developed an immunization with this strategy.^88^ In phase 2 clinical trial including 50 patients with melanoma after complete resection and at high risk of relapse, surprisingly, Chapman et al. found that a dose of Bec2 < 2.5 mg (2.5, 25, and 250 μg) was more immunogenic in melanoma than all the other doses (5 and 10 mg).^71^

Later, GD3-lactone-KLH (GD3-L-KLH) with QS-21 was compared in a sequential protocol to Bec2. The patients received Bec2/BCG at 25 μg (1 vaccination per week for 4 weeks) followed by GD3-L-KLH vaccines at 30 μg (1 vaccination per week for 4 weeks with booster 9 weeks later) or in the opposite order. The study included stage 3 or 4 melanoma patients after complete surgery of all sites. Anti-GD3 antibodies were induced only by GD3-L-KLH whether pre or post Bec2 but no immunization was observed for Bec2.^72^ Finally, in a phase 3 clinical trial, Giaccone et al. randomized Bec2 vaccine in combination with BCG therapy versus surveillance for 515 patients with localized small cells lung cancer after response to chemotherapy and radiotherapy. Median overall survivals were 16.4 and 14.3 months in the follow-up and vaccination group, respectively.^73^ The main serious adverse events were skin toxicities with one-third of grade 3. Moreover, 4% of patients presented with a grade 3 lethargy. Concerning grade 1 or 2 adverse events (AEs), skin toxicities, fever, arthralgia, lethargy, and myalgia were the most frequent. No grade 4 or 5 serious adverse events occurred in this trial related to the vaccination. Taken together, the clinical activity of Bec 2 and KLH remained limited, leading to the arrest of the vaccine development.

Ten years later, the OPT-821 vaccine targeting both GD2 et GD3 antigens with b-Glucan in adjuvant was explored for 15 young high-risk neuroblastoma (HR-NB) patients. No dose-limiting toxicity was found. At 24 months, relapse-free survival was 80%,^89^ which compared favorably with historical data showing a relapse rate of 50% and a median PFS of approximately 14 months.^90,91^ Then, in a phase 2 including 102 patients, the 2-year PFS and OS rates were 44% and 88%, respectively. Toxicities were limited to injection-related local reactions and fever. In multivariate analysis, the increase levels of IgG anti-GD2 were associated with longer PFS and OS while nor IgG neither IgM anti-GD3 increase were associated with patient survival, suggesting that anti-GD2 immunization was responsible for patient benefit.^74^ Finally, in another randomized phase 2 clinical trial including 136 patients with metastatic sarcoma, the patients received OPT-821 with KLH vaccine (anti-GM2, GD2, and GD3) versus OPT-821 monotherapy after complete resection of metastases. The adjunction of the KLH vaccine failed to improve relapse-free survival (34.5% vs. 34.8%) and OS (93.1% vs. 91.5%) compared to the adjuvant OPT-821 alone.^75^

#### Monoclonal antibodies.

—Monoclonal antibodies represent another approach to target GD3. Three main antibodies, KM-871, KW-2871 (ecromeximab), and R24 were evaluated in clinical trials, mainly for melanoma patients.

First, an IgG1 anti-GD3 (KW-2871) was tested in monotherapy during a phase 1 for 17 metastatic melanoma patients. Five doses were evaluated (1, 5, 10, 20, and 40 mg/m2). One patient had a partial response (PR) and 2 had a stable disease (SD). Importantly, no dose-limiting toxicity was observed. During this clinical trial, a second biopsy of a metastatic site was performed 10 days after vaccine initiation. In these samples, all patients presented with an increase of CD4 + lymphocyte tissue infiltration and patients with SD or PR presented with a higher increase of CD3 + and CD4 + lymphocytes compared to no responder patients.^76,77^ Another phase 1 explored the KW-2871 activity in 17 metastatic melanoma patients with increasing doses (20, 40, 60, and 80 mg/m²). The treatment doses of 40 mg/m² or higher were associated with a higher toxicity rate including grade 3 laryngospasm, chest tightness, and urticaria^92^ without a significant increase in response to lower doses. KW-2871 was also tested in association with high-dose interferon-α2b (HDI) in a phase 1/2 in 36 patients with metastatic melanoma. Cohorts 1 and 2 (5 and 10 mg/m²), with the lower dose of KW-2871, had better PFS and OS medians of 2.5 and 10.3 months, respectively. Grade 3 toxicities occurred in cohort 3 with the higher dose of 20 mg/m², including hypertension or hypotension and hypersensitivity.^78^ A phase 2 started in 2008, but the results have never been published.^93^ Taking into account these results and the safety profile, the KW-2871 clinical development was stopped due to limited clinical activity.

In parallel, a murine IgG3 R24 anti-GD3 antibody was developed and evaluated in monotherapy in 3 phase 1 clinical trials. Sixty-one patients with metastatic melanoma were included in these 2 trials: 1 patient presented with a CR and 6 patients with a PR.^79–81^ The dose of 8 mg/m² was associated with no toxicity but no response. Side effects of R24 were mainly composed of urticaria and fever. The anti-GD3 R24 antibody was also explored in combination for melanoma patients. Bajorin et al. explored the use of R24 with recombinant interleukin-2 (IL-2) in a phase 1 clinical trial including 20 patients with metastatic melanoma. Another phase 1b, explored the association of R24 with IL-2. Twenty-eight patients with metastatic melanoma were included: R24 was associated with a high toxicity rate, including cytokine-released syndrome or anaphylaxis, related to the murine origin of the antibody and the well-known toxicity of IL-2. Finally, the association of R24 with TNFα was evaluated in a phase 1 clinical trial including 8 patients with metastatic melanoma. This trial was prematurely stopped due to hemorrhagic tumor necrosis. Six patients had a PR, among them 1 patient presented an important inflammatory reaction leading to hemorrhagic and tumor lysis syndrome. Overall, in these 3 studies of combinatory strategy, the objective response rate remained very low: 8 PR were observed in 59 patients included.^82–84^ Moreover, in association with IL-2 and alpha-interferon (α-IFN), R24 showed no objective response in a phase Ib trial including 21 metastatic melanomas. Only 5 patients had a SD of more than 6 weeks (28%).^85^ Finally, a phase 1 tested the association of R24 with macrophage-colony stimulating factor (M-CSF). Three out of the nineteen patients included had a PR. The main toxicities were skin reaction and thrombocytopenia.^86^ Despite these failures, the use of R24 confirmed the induction of an infiltration of immune cells (CD4 and NK) in the tumor microenvironment following the GD3 inhibition, opening new therapeutic developments in this field with adapted therapeutic strategies.

Taken together, despite the lack of clinical activity of these vaccines and antibodies, all these studies have demonstrated the potential interest in targeting GD3, by reporting their ability to reach their target GD3, allowing the induction of immunological response and tumor microenvironment modifications. Today, however, it now seems more relevant to focus on more advanced therapeutic weapons such as antibody-drug conjugates (ADCs) or CAR-T cells.

#### Other therapeutic approaches.

—More recently, new technologies appeared to be promising strategies to target GD3 and GD3S.

First, the success of the anti-GD2 CAR-T cell development in the phase 3 clinical trial for high-risk neuroblastoma patients^94^ and in the phase 1 clinical trial dedicated to pediatric diffuse intrinsic pontine glioma,^95^ opens encouraging perspectives for anti-GD3 CAR-T cell therapeutic strategy. In the first study, 27 children with high-risk neuroblastoma were included and received a third-generation CAR T-cell therapy against GD2. Complete and PR occurred in 33% and 30% of patients, respectively. Anti-GD2 CAR-T cell administration was safe: Only 1 patient needed the infusion of rimiducid to activate an inducible caspase-9 suicide gene-reducing CAR-T cells. In the second study dedicated to refractory diffuse intrinsic pontine glioma, 3 of the 5 patients included had an objective response after anti-GD2 CAR-T cell administration with a favorable toxicity profile. In this context, the development of GD3 as CAR T-cell antigen has emerged with preclinical study exploring the activity of 2 anti-GD3 CAR T-cells from different generations with or without IL-2 administration in melanoma preclinical models. The authors observed a high complete response rate of 50% in mice treated with a second generation of anti-GD3 CAR T-cells in combination with IL-2.^96^

Other promising therapeutic strategies, including the use of ADCs or small interfering RNA (siRNA) targeting *GD3S*, are under evaluation in preclinical studies.^97,98^ ADCs anti-GD3 was also evaluated in a first clinical trial including unresectable stage 3 or 4 melanoma. Unfortunately, this trial was discontinued by Pfizer (NCT03159117).

#### Ongoing clinical trials.

—At the time of the review, no clinical trial is ongoing or recruiting concerning anti-GD3 therapies in neuro-oncology and oncology (*clinicaltrials.gov*).

## Conclusion

In conclusion, GD3 and GD3S appear to be highly expressed in glioma tissue but not in normal brain. Preclinical studies reported their involvement in glioma cell proliferation, migration, invasion, and tumor growth but also in cell cycle regulation and stemness abilities. They are also suspected to be involved in glioma microenvironment regulation. Different therapeutic approaches have been developed against GD3 and/or GD3S, alone or in combination, including direct inhibition with monoclonal antibodies or by using vaccines. These therapeutic developments were mainly performed in the melanoma field. Neuro-oncological development of GD3 inhibition represents a promising opportunity for patients.
